# Psychosocial Work Stress and Health Risks – A Cross-Sectional Study of Shift Workers From the Hotel and Catering Industry and the Food Industry

**DOI:** 10.3389/fpubh.2022.849310

**Published:** 2022-04-08

**Authors:** Bettina Hunger, Reingard Seibt

**Affiliations:** ^1^German Social Accident Insurance Institution for the Foodstuffs and Catering Industry (BGN), Government Safety Organization Foods and Restaurants, Office of Coordination Potsdam, Potsdam, Germany; ^2^Institute for Preventive Medicine of the Rostock University Medical Centre, Rostock, Germany

**Keywords:** shift work, psychosocial work stress, effort-reward imbalance, overcommitment, cardiovascular and psychological health

## Abstract

**Purpose:**

Psychosocial work stress, and shift and night work are considered risk indicators for impaired health. Using the effort-reward (ER) model, it was possible to examine which relationships exist for shift workers between clusters (CL) of different levels of psychosocial work stress and overcommitment (OC) and cardiovascular or psychological health indicators, and which predictive value is evident in individual health indicators to explain the clusters.

**Methods:**

The data were collected as part of an occupational health prevention program. The analysis sample consisted of 199 shift workers from alternating shift systems with and without night work (43%) (average age: 40 ± 12 years, men: 47%). Psychosocial work stress was recorded using the ER imbalance (ERI) questionnaire. To determine the clusters, ERI and OC were entered into a cluster analysis. Blood pressure, body mass index, waist-hip ratio, PROCAM score (risk of a heart attack within the next 10 years), sporting activity, and smoking were included as cardiovascular indicators, psychological wellbeing (GHQ-12) and inability to recovery (IR) (FABA) as psychological health indicators. Shift system, sex, and age were entered into the statistical analyses as control variables. Multinomial logistic regression models were used to identify health-related predictors to explain the ER-OC clusters.

**Results:**

Three different ER-OC clusters emerged: low-stress: 36%, normal: 44%, risk: 20%. While normal psychosocial work stress is present in the low-stress and the normal CL, in the risk CL 28% of the shift workers show a health-endangering ERI and 48% show an excessive OC. No significant cluster-specific differences were determined for the cardiovascular health indicators. Rather, the known sex and age effects were confirmed and the shift system had no significant effect. Significantly more shift workers in the risk CL had impaired psychological health (18 vs. 1/6%) and an IR (52 vs. 0/12%) than in the low-stress and normal CL. IR turned out to be the strongest predictor of the explanation for the ER-OC clusters (49%).

**Conclusion:**

IR could be assigned an independent diagnostic value for the assessment of psychosocial work stresses and discussed as a new component of occupational health screening concepts for shift workers. Independently of this, the health indicators signal an urgent need for occupational health prevention and care.

## Introduction

The effects of psychosocial work-related stress on the health of shift workers have so far been underestimated in preventive occupational health and occupational health care in Germany. Psychosocial work stress is rarely part of occupational health care programs of shift workers, although psychosocial work-related stress has been recognized as a risk factor for impaired health (1–10). In addition, shift and night work themselves can have a negative impact on health (11–20).

In stress theory observations, high work demands (effort) with low material or immaterial rewards are considered to as psychosocial work-related stress or Siegrist et al. (21) hereinafter referred to as *professional gratification crisis* or effort-reward imbalance (ERI). Experiencing such a crisis triggers intense stress reactions in those affected, which have a negative impact on health indicators and unhealthy behavior in the medium and long term, increase the risk of stress-related diseases and are intensified by excessive occupational overcommitment (OC). Individual differences in the interaction between ERI and OC have so far been largely ignored. It is assumed that shift workers who are characterized by high ERI and high OC at the same time (interaction hypothesis) have the highest risk of impaired health and impaired wellbeing (21, 22). The fact that such a stress constellation in shift workers consequently also leads to insufficient recovery has so far been neglected, as has the verification of the interaction hypothesis itself, and data on shift workers are not available.

For shift and night work, different correlations are known regarding cardiovascular (12, 14, 16–20) and psychological health indicators (11, 15), health behavior (14, 23–25) and recreational behavior (20, 26, 27). The health behavior of shift workers compared to day workers is characterized by unfavorable eating habits (14, 28, 29), more smokers (14, 29) and fewer sporting activities (23, 24, 29, 30). In addition, it is known that shift and night workers have restrictions in family and social life (13, 31, 32) and opportunities for recreation (14, 18).

Nevertheless, the percentage of shift work has increased in Germany and many other industrialized countries over the past 20 years (33, 34). In Germany, the percentage of shift workers was around 16% in 2018 (34). According to the Federal Institute for Occupational Safety and Health (35), around 20% of employees in Germany work outside the period from 07:00 to 19:00 h including 8% with staggered working hours (e.g., fixed early or late shifts), 7% in alternating shifts with night or recurring night work and 5% in alternating shifts without night work. Weekend work has also become part of everyday life for more and more employees (33).

Internationally, the definitions of shift work and night work differ widely and are often indeterminate (36). In addition, the existence of the innumerable shift systems with their different positions and lengths of working hours does not allow a uniform assessment. There is a consensus that shift work is a particular form of workload. According to the International Labor Organization, shift work is a “method of work organization that enables workers to work one after the other at the workplace, so that work processes can continue beyond individual shifts and include day and night hours” (37). Night work is defined in Germany by Section 2 and 6 of the Working Hours Act (ArbZG) and generally covers the period from 23:00 to 06:00 h with deviations. It applies to “any work that takes more than 2 h of the night” (38).

During shift work, the organism cannot adapt to the constant change in working hours, so that the shift in the circadian rhythm (sleep-wake cycle) results in restrictions on the duration and quality of sleep and has consequences for health status. Even on early and late shifts, shift workers have to work and sleep at unnatural times of the day. Since recovery deficits increase the risk of health impairments (14, 18), shift work requires balanced recovery after work.

### Shift and Night Work and Health Effects

The effects of shift and night work can be reflected in an increased activation of the sympathetic nervous system (14), which results in an increase in blood pressure (16), but also show themselves in other cardiovascular changes (e.g., inflammatory processes, blood clotting disorders) and thus influence the risk of cardiovascular diseases (12, 14). However, meta-analyses have not provided any convincing evidence for a connection between shift work and cardiovascular diseases (18, 39–41). This applies equally to cardiovascular and metabolic risk factors (17, 42). In the review by Proper et al. (42), inconsistent results on the connection between shift work and hypertension were reported. While the systematic review by Esquirol et al. (16) and the study by Stieler et al. (20) provide information on the association between shift work and hypertension, the longitudinal study by Gholami Fesharaki et al. (43) shows no correlation in this regard. Liu et al. (19) postulated in their meta-analysis that shift work is positively associated with the risk of becoming overweight and obese. Saulle et al. (44), on the other hand, did not determine such a relationship in their meta-analysis. Torquati et al. (45) postulate in their review that a non-linear association exists between shift work and cardiovascular disease that seems to show only after 5 years of shift work. After the first five years of shift work, the risk of cardiovascular events increased by 7.1% for each subsequent 5-year exposure.

There also seems to be no clear evidence of the effects of shift work on psychological health: the review by Fossum et al. (46) and the cross-sectional studies by Mauss et al. (47) and Radstaak et al. (48) confirmed no connections between shift work and indicators of wellbeing, while the longitudinal study (10-year period) by Driesen et al. (15) indicated a slight effect of shift work on the development of a depressive mood. Vallières et al. (49) assumed on the other hand, that shift workers are more prone to depression than day workers. In contrast, the survey by Bara and Arber (11) found a clear association to psychological impairments for shift workers in England. And according to the meta-analysis by Lee et al. (50), night shift work is clearly associated with an increased risk of depression.

### Psychosocial Work Stress and Health Effect

Several studies have been able to show that psychosocial (ERI-related) stress can be a risk factor for cardiovascular disease (2, 6, 7, 9) and psychological impairments (3, 5, 51, 52). Chronic stress at work is increasingly assumed to be the main cause of psychological disorders (53). The risks for this are, however, unevenly distributed in society, since socially disadvantaged population and occupational groups are more often affected by them (54).

Comparable to shift work, no consistent correlations were found between psychosocial work stress or overcommitment and increased blood pressure (hypertension). In the review by Gilbert-Ouimet et al. (4), there was a significant effect of psychosocial work stress on blood pressure in four out of seven studies, but the average increase in blood pressure was only 1 to 4 mmHg. In another five out of six studies, there was a connection between ERI and hypertension, and in two out of four studies overcommitment had a hypertensive effect. In the earlier Canadian longitudinal study by Gilbert-Ouimet et al. (1) no significant correlation between ERI and blood pressure could be found. Overcommitment, on the other hand, was associated with a significant increase in blood pressure in men and woman.

The results of the large European multi-cohort study of the IPD-Work Consortium (individual-participant data meta analysis in working populations consortium) (9) and the review by Gilbert-Ouimet et al. (4) suggest that, especially in men, there is a significant correlation between psychosocial work stress and an increased risk of cardiovascular diseases, which seems to be independent of the classic risk factors like hypertension or obesity. In the case of psychosocial work stress, an up to 1.6-fold higher risk of developing coronary heart disease or stroke was found as opposed to no work stress (10). However, this work stress risk was lower than this risk associated with the classic risk factors (i.e., smoking, high blood pressure, high serum cholesterol, obesity).

According to the meta-analysis by Rugulies et al. (8) (*n* = 84,963 employees and 2,897 new cases of depressive disorders) a consistent relationship between ERI and impaired psychological health can be assumed for both sexes. Compared to non-professionally stressed employees, an increase in the relative risk of 20 to 80 percent has been observed for ERI sufferers (25). According to Rugulies et al. (3), employees with increased psychosocial work stress are more than twice as likely to develop depressive symptoms in the next 5 years. Niedhammer et al. (5) postulate that 16% of psychological disorders can be traced back to an effort-reward imbalance.

It can be assumed that psychosocial work stress with excessive commitment of those affected also has an unfavorable effect on the psychological recovery processes in the non-working period. Psychological detachment from work during rest period is seen as a central component of individual relaxation (20, 26, 27). It is regarded as a link between working conditions and strain-related outcomes (including symptoms of fatigue and exhaustion) and is discussed as an early indicator of work-related impairments (27, 55). Richter et al. (56, 57) describe the inability to detach from work as an inability to recover. According to Wendsche and Lohmann-Haislah (58), there are differently strong correlations between psychological detachment and health indicators. There are only low correlations with cardiovascular stress indicators (20, 55).

In longitudinal studies, Sonnentag et al. (59) predict exhaustion for employees who have poor psychological detachment. And according to Siltaloppi et al. (60), employees with good recoverability suffered the least from burnout and sleep problems after 1 year. In the Finnish longitudinal study with managers (*n* = 298, three measurement times) by Feldt et al. (61), five long-term ERI-OC patterns were identified. Employees in the so-called high-risk pattern (high ERI and high OC; 20% of participants) showed poorer recovery compared to those in the low-stress pattern (normal ERI and normal OC; 24% of participants).

### Hotel and Catering Industry and Food Industry

The hotel and catering industry (HCI) and the food industry (FI) are sectors in which more than two thirds of employees do shift work - often including night work, weekend or holiday work (62, 63). Both sectors are heterogeneous branches of the economy and comprise a large number of different professions. In addition, low wages, a lack of appreciation and limited career prospects are prevalent in both sectors (64). Also, the HCI sector is characterized by long, irregular working hours which are difficult to plan, lots of overtime and, above all, high time pressure and staff shortages (63), which requires a high degree of flexibility from employees. These working conditions are perceived as exhausting and disadvantageous by more than 70% of employees (64) and often lead to problems with work-life balance (63, 65). While mainly young women (<35 years) work in the HCI sector (66, 67), 70% of the employees in the FI are older than 35 years (68). In one of the few studies on psychosocial work stress in HCI (69), 50% of the 941 hotel room cleaners reported an ERI and 60% reported poor health. In Germany too, stress at work and psychosocial risk factors have been identified in HCI (66).

### Aim of Study

In view of the work stress of working life, the question also arises in the HCI and FI as to whether certain psychosocial stress constellations increase the risk of health impairment in their shift workers. Such a study does not yet exist in German-speaking countries.

The aim of this study was therefore to use the stress-theoretical effort-reward (ER) model to clarify which relationships exist for shift workers in the HCI and FI between clusters (CL) of different levels of psychosocial work stress and overcommitment (OC) and cardiovascular or psychological health indicators, and which predictive value is evident in individual health indicators to explain the clusters. It was hypothesized that shift workers with a “risk pattern” (high ERI and OC scores) are expected to have a higher risk of decreased cardiovascular and psychological health.

## Methods

The present study is a cross-sectional study. The data from this examination were collected from 2016 to 2019 as part of an occupational health screening programme. Participation in the study was voluntary (participation rate: 75% of employees approached).

The screening programme was offered to the hotels and businesses. In the run-up to the investigation, notices and flyers were used to draw attention to the study. Immediately before the start of the study, the participants received an information letter regarding data protection, the study process and data evaluation, as well as the conditions for participation in the study. The anonymity of the data was guaranteed by transaction numbers (TANs) and a six-digit personal code.

Three hundred fifty-five employees took part in the study, 333 of which met the data quality requirements. One hundred ninety-nine shift workers, who form the database for this study, were among them. Non-shift day workers were explicitly excluded from the study because the psychosocial work stress of shift workers with and without night shifts was to be investigated in the form of easily interpretable clusters. In this approach, day workers cannot act as a control group; there is no pure cluster with only day workers. They are distributed in the clusters; such heterogeneous clusters do not contribute to explaining the relationship between different psychosocial workloads and health among shift workers.

### Survey Instruments

An occupational health survey and a cardiovascular screening programme were used as survey instruments. The survey and examinations were carried out on site in the hotels or businesses of the participants.

### Occupational Health Survey

The survey consisted of a shift work questionnaire (70) modified according to Barton et al. (71) and other standardized survey instruments as well as additional questions. Besides to socio-demographic information (e.g., sex, age, school leaving certificate, marital status, etc.), job and shift work-specific details (including job description, weekly working hours, shift system, years of work in shift work). The questionnaire also contained questions on health behavior, psychosocial work stress and overcommitment (21), as well as psychological health defined by psychological wellbeing (72) and the (in)ability to recover (56, 57). The questions about shift work formed the basis for assignment to a shift system.

Health behavior was surveyed through questions about sporting activity and smoking status. In the case of sporting activity, questions were asked about the frequency (not at all, occasionally, regularly) and the amount of time per week. For smoking, it was recorded whether someone was a smoker (YES/NO) and the average number of cigarettes smoked daily.

Psychosocial work stress was collected using the short version of the Effort-Reward Imbalance Questionnaire (ERI-Q: 21). This questionnaire allows standardized measurement of professional gratification crises and the intrinsic component of overcommitment. This version included the main scales effort (3 items, range: 3–15 points) and reward (7 items, range: 7–35 points), as well as the effort-reward ratio (ER ratio). The reward scale is made up of the three subscales job promotion, esteem and job security. Each item was measured on a five-point scale from 1 (effort: disagree, reward: agree) to 5 (effort: agree, and very distressed, reward: disagree, and very distressed). High total values for effort or reward indicate a high perception of effort or reward. The ER ratio is formed from the total values of the two main subscales using the following rule: ER ratio = ∑ effort / (∑ reward ^*^ 0.54) (21). An ER ratio of >1 indicates an effort-reward imbalance (ERI) which is said to be associated with a health risk (21). The greater the ERI (gratification crisis), the higher the health risk is.

Overcommitment (six items) was recorded with the same ERI-Q (21) on a four-point Likert scale (1 = strongly disagree to 4 = strongly agree). A total score was formed from the six items on this scale (value range: 6–24 points), according to which high values indicate a high tendency to overcommitment. The upper third of the total score is defined as the risk group (21).

Validity and Reliability of the German ERI questionnaire were rated as satisfactory. For all subscales of the short version of the ERI-Q (21) the values of the internal consistency were above 0.70 (effort: 0.74, reward: 0.79, overcommitment: 0.79). For the ER scales of the present study, Cronbach's alphas >0.70 were also determined (effort: 0.76, reward: 0.77, overcommitment: 0.81), which can be classified as acceptable or good (73).

Psychological Wellbeing was assessed by the General Health Questionnaire-12 (GHQ-12-Q: 72), which gives indications of psychological impairment or depressive symptoms. The procedure is based on a self-assessment of the wellbeing in the previous 4 weeks – in relation to normal wellbeing. The GHQ-12 contains six positively-phrased (pp) and 6 negatively-phased (np) questions. A four-point scale is used to rate the degree to which a symptom has been experienced during the last week (pp: better than usual, same as usual, less than usual, much less than usual; np: not at all, no more than usual, rather more than usual, much more than usual). Three scoring methods can be found in the literature. We used the classic binary “GHQ scoring” (0-0-1-1). The GHQ-12 total value (hereinafter GHQ score) can be between 0 and 12, whereby a higher total value is associated with increased psychological impairment (74). Based on Üstün and Sartorius (75), the cut-off value for impaired psychological health (GHQ score) is ≥5.

The validity and reliability of the GHQ-12 should be comparable to longer GHQ versions and the quality criteria should be of correspondingly high quality (Cronbach's Alphas: 0.82–0.86) (76). For the current data, Cronbach's Alpha was 0.89 and can therefore be assessed as good (73).

The inability to recover (IR) is a subscale of the questionnaire for faulty attitudes and behavioral analysis relevant to coping with work demands (FABA: 56, 57). It indicates extreme work commitment, which is associated with an accepted limited ability to recover in the sense of an inefficient coping style. IR was recorded with six items on the basis of a four-level ranking scale (1 = does not apply at all to 4 = applies very much). Then, the IR score (range: 6–24 points) was formed over the six items, which can be assigned using percentile values to normal (6–18 points), noticeable (19–21 points) and very noticeable (22–24 points) recovery values.

The reliability of the subscale inability to recover is given with a Cronbach's alpha of 0.79 (57). In the present study, a Cronbach's alpha of 0.86 was determined for the IR score, which can also be assigned to the good range (73).

### Cardiovascular Screening Programme

Cardiovascular health was studied using the following indicators:

- home blood pressure monitoring.
- body measurements (body-mass-index, waist-hip-ratio).
- PROCAM score [Prospective Cardiovascular Münster score (77)].

Blood pressure (BP) measurement was carried out as a self-measurement on 4 days in accordance with the guidelines of the European Society of Cardiology (ESC) and the European Society of Hypertension (ESH) (78). BP was measured on the upper arm and while sitting; a BOSO medicus (Bosch + Sohn GmbH, Jungingen, Germany) measuring device was used to measure BP. The participants were instructed to take six BP measurements daily between 06:00 and 10:00 h, at intervals of 2–3 h after 3 min of rest (total of *n* = 24 measurements). BP mean values were calculated from these 24 measured values and used to determine BP status (hypertensives ≥135/85 mmHg, normotensives <135/85 mmHg (78). In addition, it was determined whether antihypertensive drugs were being taken. Shift workers taking antihypertensive medications were classified *per se* as hypertensives and considered separately in BP-related analyses.

Body measurements serve to estimate the fat distribution pattern in the body and represent important determinants for the health risk in the case of being overweight or obese (24, 79, 80). Body weight and height as well as waist and hip circumference were measured for all participants. The body-mass-index (BMI) and waist-hip-ratio (WHR) were calculated from these values using the appropriate formulas:


$$\begin{array}{l} \text{BMI}=\frac{\text{body weight }\left[\text{kg}\right]\;}{\text{ body lengthe }\left[{\text{m}}^{2}\right]}. \end{array}$$

According to the criteria of the German Adiposity Society (81), normal weight (18.5–24.9 kg/m^2^), overweight (25.0–29.9 kg/m^2^) and obesity (≥30 kg/m^2^) can be determined using the BMI.


$$\begin{array}{l} \text{WHR}=\frac{\text{waist circumference }\left[\text{cm}\right]}{\text{ hip circumference }\left[\text{cm}\right]}. \end{array}$$

WHR values above 1.0 in men and above 0.85 in women indicate abdominal obesity (82), which is associated with an increased risk of a metabolic syndrome (83).

PROCAM score estimates the individual risk of suffering a heart attack (myocardial infarction) in the period of the next 10 years. It is based on the epidemiological PROCAM Study (*Prospective Cardiovascular Münster*) and applies to women and men aged 20–75 years. Here, the rapid test was used to calculate cardiovascular health, which was introduced as a simplification of the precise PROCAM score (84) and did not record blood parameters (cholesterol, triglycerides) (77). The following eight classic risk factors are included in the calculation of the simplified PROCAM score, each of which contributes independently to the individual heart attack risk: age, sex, systolic BP, BMI, anamnestic data on antihypertensive drugs, diabetes mellitus, smoking habits and any family history of heart attacks.

Depending on the severity, different point values are assigned for the risk factors and then added to the PROCAM total score (hereinafter only PROCAM score). The score can be between 0 and 59 for men and between 0 and 56 for women. The lower the PROCAM score, the better the cardiovascular health of a shift worker is. In addition, the risk of heart attack risk can be assessed using an evaluation table by Assmann et al. (84): if the result is in the green range, there is a low risk of heart attack (<10%) over the next 10 years, in the yellow range there is a medium risk (10–20%) and in the red range a high risk (>20%).

### Statistical Analyses

The statistical analysis of the data was carried out using *Statistical Package for the Social Science* (SPSS INC, Chicago, IL, USA) for Windows (Version 27).

The focus of this article is on the comparison of clusters with different psychosocial work stress and their relationship to indicators of cardiovascular and psychological health. First, the health indicators for the clusters were descriptively analyzed (mean values, standard deviations, medians, quartiles). In addition to shift work, sex and age were included as control variables in all statistical analyses (exception: PROCAM score).

In order to identify differences (mean value comparisons) between the cardiovascular and psychological indicators within the ER-OC clusters, univariate covariance analyses were carried out with the control variables. The Chi^2^ test was used to test the difference between categorical variables.

Multinomial logistic regression analyses were carried out to examine the relationship of the cardiovascular and psychological health indicators (independent variables) as well as the control variables on the ER-OC clusters (criterion variable). For this purpose, the individual health indicators or control variables were correlated with the ER-OC clusters in the first step of the analysis (Spearman rank correlation) and evaluated according to the recommendations of Bühl (85). The influence of the control variables on these relationships was adjusted using partial correlations (85). Correlation coefficients r ≤ ±0.10 are interpreted as being independent of one another.

A probability of error of α <0.05 was specified as a statistical significance criterion and supplemented by effect sizes. The interpretation of the effect sizes was based on the conventions of Cohen (86). In the analysis of variance and in the Chi^2^ test, small effect sizes from $\eta_{\text{partial}}^{2}$ ≥0.01 or d ≥0.20 were considered statistically significant effects.

A *post-hoc p*ower analysis was performed to assess the significance of the study (statistical power) using the G^*^Power program (87). This analysis was calculated for a univariate ANOVA with main effects (three groups). Given the total sample size (*n* = 199), the alpha error probability (α err prob = 0.05), a medium effect size (f = 0.25), and a number of degrees of freedom (df = 2), this analysis returns an actual power (1 – β err prob) of 0.90, and an error probability of the test (β) of 0.10, respectively. With an effect size of η^2^ = 0.06 (corresponding to an f of about 0.25) and a power of 0.90, one would need 68 subjects per cluster (204 in total) to obtain a significant result with a one-factor ANOVA (α <0.05).

### Determination of the Clusters With Different Psychosocial Work Stress

Groups of shift workers with different psychosocial work stress experiences were identified using a two-step cluster center analysis of the z-transformed values (85) of the effort-reward ratio (ER ratio) and overcommitment (OC) (ER-OC clusters). There were 199 complete records of shift workers included in the cluster analysis. Based on theoretical considerations, a three-cluster solution with the following well-interpretable clusters (CL) was favored (Table 1): low-stress cluster (LC: *n* = 71, 36%), normal cluster (NC: *n* = 88, 44%) und risk cluster (RC: *n* = 40, 20%).

**Table 1:** Characteristic of effort-reward (ER) subscales and overcommitment (OC) in the ER-OC clusters (CL).

**Characteristic**	**Low-stress CL** **(*n* = 71)**	**Normal CL** **(*n* = 88)**	**Risk CL** **(*n* = 40)**	**Test size**	***p*-Value**	**Effect size**
	Means ± standard deviation					$\eta_{\text{partial}}^{2}$
Effort [3–15 pts]	5.9 ± 2.0	9.3 ± 1.7	10.3 ± 2.8	81.81	<0.001^***^	0.458
Reward [7–35 pts]	30.4 ± 3.7	26.3 ± 4.2	24.2 ± 5.1	29.11	<0.001^***^	0.231
-Job promotion [3–15 pts]	12.1 ± 2.1	10.3 ± 2.4	9.7 ± 2.9	13.92	<0.001^***^	0.125
-Esteem [2–10 pts]	8.6 ± 1.7	7.1 ± 1.9	6.3 ± 1.8	23.59	<0.001^***^	0.196
-Job security [3–15 pts]	9.6 ± 0.9	8.9 ± 1.4	8.2 ± 2.0	14.05	<0.001^***^	0.126
ER ratio	0.4 ± 0.2	0.7 ± 0.2	0.8 ± 0.3	81.48	<0.001^***^	0.475
Overcommitment (OC) [63–24 pts]	9.9 ± 2.3	13.7 ± 1.9	18.5 ± 2.0	215.58	<0.001^***^	0.690
Classification	Frequency (number)					d
-ER ratio >1	% (n)	3.4 (3)	27.5 (11)	32.77	<0.001^***^	0.888
-OC >18	% (n)	1.1 (1)	47.5 (19)	77.72	<0.001^***^	1.601

*pts, points. High values of the subscales mean high effort, high reward or high overcommitment. % (n): frequency in percent (number of shift workers). Chi-square test (test size: χ^2^-value, effect size: d); covariance analyses (design: constant term + ER-OC cluster, test size: F-value, effect size: $\eta_{partial}^{2}$: partial eta-square); p-value: significance (two-sided)*:

** p < 0.01.

*** *p < 0.001*.

*Effect size according to Cohen (86): d: <0.20 = no effect, 0.20–0.49 = small effect, 0.50–0.79 = medium effect, ≥0.80 = large effect; $\eta_{partial}^{2}$: <0.01 = no effect, 0.01–0.05 = small effect, 0.06–0.14 = medium effect, >0.14 = large effect*.

Compared to those in the normal and high-risk cluster, the shift workers in the low-stress cluster showed on average the significantly (*p* <0.001) lowest effort (∅ 6 points), the highest reward (∅ 30 points) and the lowest overcommitment (∅ 10 points). Conversely, the risk cluster stands out on average due to the highest effort (∅ 10 points), the lowest reward (∅ 26 points) and the highest overcommitment (∅ 19 points). The average values of the three subscales of the normal cluster are in each case between the low-stress and risk cluster, but differ significantly from both clusters (*p* = 0.027– <0.001). It is assumed that (normal) psychosocial work stress without health risk exists in the low-stress cluster and in the normal cluster. In contrast, more than a quarter (28%) of shift workers are in the risk cluster with a health-endangering effort-reward imbalance and around half (48%) with an excessive propensity to overcommitment (Table 1). Almost every fifth shift worker in the risk cluster (18%) is at risk from both psychosocial work stress and excessive effort.

### Sample

The sample consisted of 199 shift workers from the hotel and catering industry (HCI) as well as the food industry (FI) affiliated by the German Government Safety Organization Foods and Restaurants (BGN). About half of it was made up of female (53%) and male shift workers (47%) with an average age of 41 ± 11 years; all shift workers were employed full-time. More than half of the shift workers were younger than 40 years and 22% were older than 50 years. The distribution of the sexes (*p* = 0.172) and the age of the shift workers (*p* = 0.411) did not differ in the three ER-OC clusters (Table 2).

**Table 2:** Socio-demographic data in the ER-OC clusters (CL).

**Personal und job-related characteristic**	**Dimension**	**Low-stress CL** **(*n* = 71)**	**Normal CL** **(*n* = 88)**	**Risk CL** **(*n* = 40)**	**Test size**	***p*-Value**	**Effect size**
Age [years]	M ± SD	41.2 ± 12.4	38.7 ± 11.3	39.6 ± 10.9	0.89^++^	0.411	0.009
- <40	% (n)	53.4 (39)	58.0 (51)	52.5 (21)	1.50	0.827	0.129
-40–50	% (n)	20.5 (15)	21.6 (19)	27.5 (11)			
->50	% (n)	26.0 (19)	20.5 (18)	20.0 (8)			
Working time [hours/week]	M ± SD	39.1 ± 3.3	40.6 ±3.8	43.0 ± 7.2	11.3^++^	0.001^***^	0.104
Shift work [working years]	M ± SD	10.1 ± 8.2	9.8 ± 7.6	10.3 ± 7.4	0.05^++^	0.950	0.001
Sex
-Men	% (n)	49.3 (35)	51.1 (45)	32.5 (13)	4.13^+^	0.127	0.291
-Women	% (n)	50.7 (36)	48.9 (43)	67.5 (27)			
Shift system
-Permanent shift system	% (n)	4.2 (3)	8.0 (7)	12.5 (5)	2.59^+^	0.629	0.230
-Alternating shift without night work	% (n)	50.7 (36)	50.0 (44)	47.5 (19)			
-Alternating shift with night work	% (n)	45.1 (32)	42.0 (37)	40.0 (16)			
School graduation
-Lower secondary education	% (n)	22.5 (16)	15.9 (14)	10.0 (4)	6.61^+^	0.359	0.371
-Middle secondary education	% (n)	54.9 (39)	63.6 (56)	65.0 (26)			
-Upper secondary education	% (n)	18.3 (13)	19.3 (17)	25.0 (10)			
-Other school education	% (n)	1.1 (1)	1.1 (1)	— (—)			

*M ± SD, mean ± standard deviation; % (n), frequency in percent (number of shift workers). ^+^ = Chi-square test (test size: χ^2^-value, effect size: d); ^++^ = covariance analyses (design: constant term + ER-OC cluster, test size: F-value, effect size: $\eta_{partial}^{2}$); p-value: significance (two-sided)*:

*** *p < 0.001*.

*Effect size according to Cohen (86): d: <0.20 = no effect, 0.20–0.49 = small effect; $\eta_{partial}^{2}$: <0.01 = no effect, 0.01–0.05 = small effect, 0.06–0.14 = medium effect, >0.14 = large effect. Corrected R-squared: age = 0.020, working time = 0.111, shift work = 0.005*.

However, the average weekly working time in the ER-OC clusters was significantly different (*p* = 0.001, medium effect): With an average of 39 hours/week, the shortest working time was found in the low-stress cluster and the longest working time with 43 hours/week in the risk cluster. On average, the employees have been working in shifts for 10 years, most of them in an alternating shift system (93%), only a small proportion (7%) in a permanent shift system. For 50% of employees, the alternating shift system consisted of a morning (start: 06:00 to 09:00 h, end: 02:00 to 05:00 h) and afternoon shift ((start: 06:00 to 09:00 h, end: 02:00 to 05:00 h) and afternoon shift (start: 12:00–03:00 h, end: 08:00–11:00 h with a small percentage of night shift), 43% worked in a forward-rotating, three-shift system with early (06:00–02:00 h), late (02:00–10:00 h) and night shift (10:00–6:00 h).

The school education of shift workers did not differ in the three ER-OC clusters (*p* = 0.413) [German secondary schools are divided into three main categories: lower-level secondary school (*Hauptschule*), middle-level secondary school (*Realschule*) and upper-level secondary school (*Gymnasium*)]: 17% of participants indicated lower-level secondary education and 61% middle-level secondary education, and 20% had completed upper-level secondary education.

The field of activity of the participants was manifold. These activities have been assigned into six job categories and are composed of kitchen staff (12%), restaurant and hotel specialists (18%), line/plant operators (22%) as well as employees in warehouse logistics/packaging (18%), food production and food sales (15%) and other service areas (15%) (e.g., maintenance, house services, security, cleaning staff). There were no statistically significant differences between the ER-OC clusters for the individual areas of activity (*p* = 0.061). From this range of activities, around a third could be assigned to the predominantly physical (41%), a quarter to the predominantly psychological (21%) and almost half to the “mixed” (38%) work stress (*p* = 0.187).

## Results

To detect differences in cardiovascular and psychological health indicators between ER-OC clusters, univariate analyses of covariance were performed with the control variables (shift system, sex, age; exception: PROCAM score).

### Psychosocial Work Stress and Cardiovascular Health

For the cardiovascular indicators (exception: diagnosis of hypertension), no statistically significant differences (*p* > 0.05) were found between the three ER-OC clusters (Table 3). The trend, however, shows that the cardiovascular indicators are more unfavorably pronounced in the low-stress cluster than in the risk cluster. There are no or only very slight correlations between the cardiovascular indicators and the ER-OC clusters (R = −0.07 to −0.13).

**Table 3:** Main effects and classification of cardiovascular indicators in the ER-OC clusters (CL).

**Cardiovascular indicator**	**Dimension**	**Low-stress CL** **(*n* = 71)**	**Normal-CL** **(*n* = 88)**	**Risk-CL** **(*n* = 40)**	**Test size**	***p*-Value**	**Effect size**	**R^**2**^-value**
**Blood pressure (BP)**
- Systolic BP [mmHg]	M ± SD	135.3 ± 12.3	131.4 ± 10.2	132.9 ± 16.8	2.16^++^	0.118	0.022	0.178
- Diastolic BP [mmHg]	M ± SD	83.6 ± 8.5	81.1 ± 8.1	82.5 ± 11.3	1.31^++^	0.272	0.013	0.079
- Hypertension	% (n)	64.8 (46)	44.3 (39)	50.0 (20)	6.76^+^	0.034*	0.375	
- Taking antihypertensives	% (n)	12.7 (9)	15.9 (14)	10.0 (4)	0.89^+^	0.640	0.134	
**BP without antihypertensives**
- Systolic BP [mmHg]	M ± SD	134.3 ± 12.6	131.2 ± 10.4	132.6 ± 12.9	1.15^++^	0.320	0.014	0.173
- Diastolic BP [mmHg]	M ± SD	82.9 ± 8.7	81.0 ± 8.4	82.1 ± 11.7	0.53^++^	0.591	0.006	0.058
- Hypertension	% (n)	59.7 (37)	43.2 (32)	47.2 (17)	3.78^++^	0.151	0.278	
**Body measurements**
Body-mass-index [kg/m^2^]	M ± SD	26.6 ± 4.0	26.5 ± 4.3	25.8 ± 3.8	0.22^++^	0.799	0.002	0.035
- Normal weight	% (n)	38.0 (27)	36.4 (32	52.5 (21)	6.95^+^	0.326	0.381	
- Overweight	% (n)	39.4 (28)	45.5 (40)	32.5 (13)				
- Obesity	% (n)	22.5 (16)	18.2 (16)	15.0 (6)				
Waist circumference [cm]	M ± SD	92.2 ± 13.2	90.5 ± 13.3	87.5 ± 10.5	0.56^++^	0.575	0.006	0.252
- Normal risk	% (n)	40.8 (29)	48.9 (43)	55.0 (22)	3.10^+^	0.542	0.252	
- Increased risk	% (n)	19.7 (14)	21.6 (19)	15.0 (6)				
- Significantly increased risk	% (n)	39.4 (28)	29.5 (26)	30.0 (12)				
Waist-hip-ratio	M ± SD	0.90 ± 0.11	0.88 ± 0.10	0.86 ± 0.11	0.34^++^	0.715	0.003	0.180
- Normal risk [♂ ≤ 1.0, ♀: ≤ 0.85]	% (n)	69.0 (49)	79.5 (70)	75.0 (30)	2.32^+^	0.314	0.217	
- Increased risk [♂: >1.0, ♀: >0.85]	% (n)	31.0 (22)	20.5 (18)	25.0 (10)				
PROCAM score [pts]
Heart attack risk (*n* = 152)	M ± SD (n)	18.0 ± 9.6 (62)	16.2 ± 10.2 (74)	15.1 ± 7.1 (36)	0.90^++^	0.409	0.011	0.305
- Low (<10%)	% (n)	92.7 (51)	87.9 (58)	96.8 (30)	7.38^+^	0.117	0.452	
- Medium (10–20%)	% (n)	1.8 (1)	10.6 (7)	3.2 (1)				
- Large (>20%)	% (n)	5.5 (3)	1.5 (1)	— (—)				
**Health behavior**
Sporting activity
- Sport (no)	% (n)	46.5 (33)	43.2 (38)	35.0 (14)	1.39^+^	0.499	0.168	
- Sport (yes – regularly)	% (n)	43.7 (31)	40.9 (36)	37.5 (15)				
- Sport (yes – occasionally)	% (n)	9.9 (7)	15.9 (14)	27.5 (11)				
- Sport (yes – time [hours/week])	M ± SD (n)	2.7 ± 2.2 (38)	2.8 ± 2.5 (50)	2.1 ± 1.7 (26)	0.71	0.492	0.013	0.010
Smoking status
- Non-smoker	% (n)	45.1 (32)	61.4 (54)	55.0 (22)	4.21^+^	0.122	0.294	
- Smoker	% (n)	54.9 (39)	38.6 (34)	45.0 (18)				
- Smoker – cigarettes [number/day]	M ± SD (n)	12.3 ± 5.2 (39)	13.1 ± 7.0 (34)	13.4 ± 6.3 (18)	0.38^++^	0.687	0.088	0.015

pts, points; M ± SD: mean ± standard deviation; % (n): frequency in percent (number of shift workers). ^+^ = Chi-square test according to Pearson (test size: χ^2^-value, effect size: d); ^++^ = covariance analyses (design: constant term + ER-OC cluster + shift system + sex + age, test size: F-value, effect size: $\eta_{partial}^{2}$); p-value: significance (two-sided): *p <0.05. Effect size according to Cohen (86): d: <0.20 = no effect, 0.20–0.49 = small effect; $\eta_{partial}^{2}$: <0.01 = no effect, 0.01–0.05 = small effect, 0.06–0.14 = medium effect. R^2^, corrected R-squared.

Essential, significant main effects, however, occur for blood pressure (BP) and body measurements in the classic control variables sex (*p* = 0.008- <0.001; $\eta_{\text{partial}}^{2}$ = 0.038–0.197, small to large effects) and age (*p* = 0.004- <0.001; $\eta_{\text{partial}}^{2}$ = 0.012–0.122, small to medium effects); the shift system has no effect on any indicators (*p* > 0.05) (Table 4).

**Table 4:** Main effects of covariates of cardiovascular indicators in the ER-OC clusters (CL) (*n* = 199).

**Cardiovascular indicator**	**Shift system**			**Sex**			**Age [years]**		
	**Test size**	***p*-Value**	** $\eta_{partial}^{2}$ **	**Test size**	***p*-Value**	** $\eta_{partial}^{2}$ **	**Test size**	***p*-Value**	** $\eta_{partial}^{2}$ **
**Blood pressure (BP) taking antihypertensives**
- Systolic BP [mmHg]	0.01	0.959	<0.001	34.82	<0.001^***^	0.153	12.36	<0.001^***^	0.060
- Diastolic BP [mmHg]	1.25	0.264	0.006	8.17	0.005^**^	0.041	13.48	<0.001^***^	0.041
**BP without taking antihypertensives**
- Systolic BP [mmHg]	0.10	0.750	0.001	33.02	<0.001^***^	0.166	7.94	0.005^**^	0.046
- Diastolic BP [mmHg]	1.01	0.316	0.006	7.18	0.008^**^	0.041	8.74	0.004^**^	0.050
**Body measurements**
Body-mass-index [kg/m^2^]	2.69	0.103	0.014	7.57	0.006^**^	0.038	2.35	0.127	0.012
Waist circumference [cm]	0.08	0.783	<0.001	47.21	<0.001^***^	0.197	26.84	<0.001^***^	0.122
Waist-hip-ratio	1.21	0.272	0.006	29.42	<0.001^***^	0.132	24.17	<0.001^***^	0.111
PROCAM score [pts]^++^	3.85	0.051	0.019	—	—	—	—	—	—
**Health behavior**
Sporting activity^+^	3.19^+^	0.076	0.016	2.21^+^	0.138	0.011	0.36^+^	0.552	0.002
Sport (yes) – time [hours/week]	0.04	0.845	<0.001	1.71	0.194	0.016	0.03	0.861	<0.001
Smoking status^+^	4.15^+^	0.042*	0.016	1.84^+^	0.177	0.009	0.06^+^	0.805	<0.001
Smoker – cigarettes [number/day]	0.01	0.909	<0.001	1.89	0.172	0.022	1.00	0.321	0.012

*pts, points. ^+^ = Chi-square test (test size: χ^2^-value, effect size: d); covariance analyses (design: constant term + ER-OC cluster + shift system + sex + age, test size: F-value, effect size: $\eta_{partial}^{2}$); p-value: significance (two-sided)*:

** *p < 0.01*,

*** *p < 0.001*.

*Effect size according to Cohen (86): d: <0.20 = no effect; $\eta_{partial}^{2}$: 0.01–0.05 = small effect, 0.06–0.14 = medium effect. ^++^ The PROCAM score included sex and age as risk factors*.

The effects on health behavior are reversed: no sex or age effects (*p* > 0.05) can be determined, but a small shift system effect for smoking (*p* = 0.042; d = 0.210). There are significantly more smokers in the alternating shift system with night shifts (53%) than in those without night shifts (41%) or the permanent shift system (33%) (Table 4).

It could be confirmed that the BP values in men are significantly higher than in women (mean: 138/84 vs. 129/81 mmHg, $\eta_{\text{partial}}^{2}$ = 0.118/0.022, medium/small effect), and that BP increases with age (<40 vs. >50 years, mean: 131/81 vs. 137/84 mmHg, $\eta_{\text{partial}}^{2}$ = 0.027/0.038, small effects); it explains 18% of the variance in systolic and 8% in diastolic BP. The same sex and age effects were recorded for WC, BMI, WHR and PROCAM score, with the explanation of variance varying between 3 (BMI) and 30% (PROCAM score) (Tables 3, 4). Regardless of this, more than half of the shift workers have hypertonic BP (54%) and are overweight or obese (60%). Elevated values for the waist-hip ratio are found in a quarter (25%) of shift workers. 5% of shift workers have a medium risk and 2% a high risk of having a heart attack in the next 10 years. In addition, only 41% of them participate in regular physical activities (sport) and 46% are smokers; they smoke an average of 13 cigarettes a day (Table 3).

### Psychosocial Work Stress and Psychological Health

For psychological health, there are significant differences between the ER-OC clusters — both for psychological wellbeing (*p* <0.001; $\eta_{\text{partial}}^{2}$ = 0.069, medium effect) as well as for inability to recover (IR) (*p* <0.0001; $\eta_{\text{partial}}^{2}$ = 0.430, large effect). Taking the control variables into account, psychological wellbeing explains 9% and the inability to recover 44% of the variance in the ER-OC clusters (Table 5).

**Table 5:** Main effects of psychological indicators and covariates and classification of psychological indicators in the ER-OC clusters (CL).

**Psychological indicators**	**Dimension**	**Low-stress CL** **(*n* = 71)**	**Normal CL** **(*n* = 88)**	**Risk CL** **(*n* = 40)**	**Test value**	***p*-Value**	**Effect size**
**Psychological wellbeing**	M ± SD	0.4 ± 1.0	0.8 ± 1.7	1.9 ± 3.4	7.16^++^	0.001^***^	0.069
[GHQ score: 0–12 pts]	M (Q_25_, Q_75_)	0.0 (0, 0)	0.0 (0, 1)	0.0 (0, 2)			
Covariate
- Shift system					19.89^+^	<0.001^***^	0.093
- Sex					4.80^+^	0.030^*^	0.024
- Age (years)					1.82^++^	0.179	0.009
Classification GHQ score
- Normal [0–4 pts]	% (n)	98.6 (70)	94.3 (83)	82.5 (33)	11.04^+^	0.004^**^	0.485
- Impaired [5–12 pts]	% (n)	1.4 (1)	5.7 (5)	17.5 (7)			
**Inability to recover (IR)**	M ± SD	11.3 ± 2.8	14.7 ± 3.1	18.5 ± 3.2	73.30^++^	<0.001^***^	0.430
[IR score: 6–24 pts]						
Covariate
- Shift system					0.55^+^	0.459	0.003
- Sex					3.98^+^	0.047^*^	0.020
- Age (years)					2.28^++^	0.133	0.012
Classification IR score
- Normal [6–18 pts]	% (n)	100.0 (71)	87.5 (77)	47.5 (19)	61.10^+^	<0.001^***^	1.331
- Noticeable [19–20 pts]	% (n)	— (—)	9.1 (8)	17.5 (7)			
- Very noticeable [≥21 pts]	% (n)	— (—)	3.4 (3)	35.0 (14)			

*pts, points; M ± SD, mean ± standard deviation; M (Q_25_, Q_75_), median and quartile; % (n), frequency in percent (number of shift workers). ^+^ = Chi-square test (test size: χ^2^-value, effect size: d); ^++^ = covariance analyses (design: constant term + ER-OC cluster + shift system + sex + age, test size: F-value, effect size: $\eta_{\text{partial}}^{2}$); p-value: significance (two-sided)*:

* *p < 0.05*,

** *p < 0.01*,

*** *p < 0.001*.

*Effect size according to to Cohen (86): d: 0.50–0.79 = medium effect, ≥0.80 = large effect; $\eta_{\text{partial}}^{2}$: <0.01 = no effect, 0.01–0.05 = small effect, 0.06–0.14 = medium effect. ^+^ The PROCAM score included sex and age as risk factors. Corrected R-squared: GHQ score = 0.093, IR score = 0.444*.

According to Üstün and Sartorius (75) a GHQ score of five points or more shows indications of psychological impairment. This affected 7% of all shift workers, only 1% in the low-stress cluster, but 18% in the risk cluster (*p* = 0.004; d = 0.485; small effect).

Sex and shift system have a significant influence on psychological wellbeing (*p* = 0.030- <0.001; $\eta_{\text{partial}}^{2}$ = 0.024–0.093, small to medium effects), but not age (*p* = 0.179). Psychological impairment is significantly more common in women than in men (10 vs. 2%: *p* = 0.019, d = 0.337, small effect) and shift workers who do night shifts report significantly more conspicuous GHQ scores (12%) and thus depressive symptoms than those from the other two shift systems (0–3%: *p* = 0.033, = 0.378, small effect).

Even in the case of inability to recover, the most favorable recovery values (∅ 11 points) are shown in the low-stress cluster and the most unfavorable recovery values (∅ 19 points) in the risk cluster, whereby the cluster mean value is at the limit of the conspicuous area. Accordingly, all shift workers in the low-stress cluster reported normal recovery values, but only 48% in the risk cluster (*p* <0.001, d = 1.33, large effect). The severity of the inability to recover is only influenced by sex (*p* = 0.047, $\eta_{\text{partial}}^{2}$ = 0.020, small effect), not by age and shift system (p > 0.05). On average, women indicate significantly higher IR scores than men (∅ 15 vs. 13 points: p = 0.021, $\eta_{\text{partial}}^{2}$ = 0.027, small effect).

### Relationship of Health Indicators to ER-OC Clusters

The correlation analyses of the examined health-related indicators confirmed that there is no or only a very low correlation (R = −0.14–0.10) between the cardiovascular health indicators (BP, BMI, WHR, PROCAM score, sport activities (hours/week), smoking status) and the ER-OC clusters. For the psychological health variables, the ER-OC clusters also yielded only a low correlation to the GHQ score (R = 0.24) and a medium correlation to the IR score (R = 0.66). This is also confirmed by the partial correlation coefficients. Psychological well-being (GHQ score) and inability to recover (IR score) were significantly decreased in the risk cluster. There were no correlations of the control variables shift system (R = −0.03), sex (R = 0.10), and age (R = −0.07) with the ER-OC clusters, i.e., they did not contribute to the explanation of the ER-OC cluster.

Since a total model with cardiovascular indicators and control variables would not yield significant effects, these variables were omitted from the multinomial logistic regression model. Thus, only the variables GHQ score and IR score were entered into the multinomial logistic regression model, with the low-stress cluster being used as a reference category. Table 6 summarizes the effects of the indicators estimates of each predictor.

**Table 6:** Regression model of psychological indicators to explain ER-OC clusters in shift workers (*n* = 199).

**Model**	**Parameter estimates**						
	**Regression**	**SE**	**Wald test**	**p-value**	**Exp(B)**	**95% confidence interval for B**	
	**coefficient B**					**Lower bound**	**Upper bound**
**Normal cluster**
(constant)	−5.05	0.94	28.75	<0.001^***^			
GHQ-score	0.11	0.14	0.63	0.427	1.115	0.85	1.46
IR-score	0.39	0.07	29.22	<0.001^***^	1.484	1.29	1.71
**Risk cluster**
(constant)	−13.00	1.72	57.23	<0.001^***^			
GHQ-score	0.31	0.15	4.36	0.037^*^	1.367	1.02	1.83
IR-score	0.80	0.11	54.79	<0.001^***^	2.217	1.80	2.74

*SE, standard error. Dependent variable: ER-OC cluster, independent variables (predictors): GHQ-score, IR-score. Multinomial logistic regression (method: enter), number of degrees of freedom: 1, reference category: low-stress cluster (n = 71). P-value: significance (two-sided)*:

* *p < 0.05*,

*** *p < 0.01*.

The regression model contains a significant explanatory component (χ^2^(4) = 119.73, *p* <0.001). Psychological wellbeing (GHQ score) and inactivity to recovery (IR score) were confirmed as predictors of the level of psychosocial work stress in overcommitted shift workers. Both variables explain 52% of the variance of the ER-OC clusters. Thereby, the GHQ score alone explains 8% and the IR score alone 49% of the variance of the ER-OC clusters. However, both variables appear to have different relevance for the normal and risk clusters.

Whereas, in the normal cluster only the IR score turned out to be a predictor for the ER-OC clusters, in the risk cluster, the GHQ score, in addition to the IR score, also showed to be a significant predictor. Compared to the low-stress cluster, the chance of belonging to the normal cluster increases by almost 50% when the IR score increases by one point. As expected, this effect is more pronounced in the risk cluster: If GHQ and IR score each increase by one point, the relative probability of a shift worker of belonging to the risk group increases by 1.4 and 2.3 times, respectively (Table 6). About 70% of all shift workers are assigned to the correct ER-OC cluster using this model.

## Discussion

For the shift workers in the hotel and catering industry and food industry, a three-cluster solution was found using the *model of the professional gratification crisis* (21), in which a risk cluster was shown in addition to a low-stress and normal cluster. In shift work, this cluster is characterized by a specific work-related risk constellation (high ERI and high OC at the same time) for an increased risk of psychological health impairments (depressive symptoms), but above all inadequate recovery. In the low-stress cluster there are neither shift workers with an imbalance of effort and reward, nor people with high overcommitment, in the normal cluster there is only a small proportion (ERI: 3%, OC: 1%).

In addition, the shift workers in the low-stress cluster worked an average of 4 h a week less than those in the risk cluster. The mean values of the GHQ and IR scores in the low-stress and normal clusters initially indicate good psychological health; they explain 45% of the variance of the ER-OC clusters. Inability to recover turned out to be the strongest predictor (explained variance: 43%) and is interpreted as a new finding in the context of the *model of the professional gratification crisis* (21). In contrast, the individual cardiovascular indicators (BP, BMI, WHR, PROCAM score, sport activities (time/week), smoking status) each explain less than one percent of the variance of the ER-OC clusters. This supports the assumption of Cottini (88) that psychosocial work stress affects psychological health rather than physical health. It is known that a lower social status is associated with a higher risk of many physical and psychological illnesses (89). However, it is possible that shift workers with physical health problems stoped working in the hotel and catering industry and food industry and left shift work.

Rather, the known significant main effects for sex and age were confirmed for the cardiovascular indicators, according to which, in particular, more men than women indicated health-endangering cardiovascular risks. The shift system had almost no influence on the cardiovascular indicators. There was only a small shift system effect for smoking.

For psychological wellbeing and inability to recover there was also a small significant effect for sex: women were more psychologically impaired and reported less favorable ability to recover than men (GHQ score ≥5: 10 vs. 2%; IR score: 18 vs. 14%). In addition, 12% of the shift workers in the alternating shift system with night shift showed impaired psychological health and only 3% of the shift workers without night work (shift system effect). This confirms the already known effect that shift systems with night work in particular seem to increase the risk of depressive symptoms (50).

Conforming to the interaction hypothesis of the ERI model (21, 22) and the research literature (1, 3–5) 18% of the shift workers with increased psychosocial work stress and a strong tendency to overcommit (risk cluster) reported impaired psychological wellbeing, while this only affected 1% in the low-stress cluster and 6% in the normal cluster. Overall, 7% of all shift workers reported depressive symptoms. Comparative data on the prevalence of depressive symptoms in the general adult population from Germany are only indirectly available through the *German Health Interview and Examination Survey for Adults (DEGS1 study)* (90), because the German version of the Patient Health Questionnire (PHQ-9) in the study was used to record depressive symptoms (91, 92). Depressive symptoms (PHQ-9 ≥10 points) existed here (age range: 18–59 years) for 9% of the adults. Thus, the share of psychologically impaired shift workers is initially comparable with that of the general German population (90, 93). In the risk cluster, however, this proportion is twice as high (18%) and somewhat lower in the normal cluster (6%) than in this population.

The fact that the inability to recover (56, 57) or the inability to “psychologically detach from work” (55) functions as the most important predictor to explain the ER-OC clusters is an new aspect in the context of the ERI model (21). In the risk clusters, more than half of the shift workers (52%) noticed that they had poor recovery ability, while in the low-stress cluster the phenomenon of inability to recover did not occur at all and in the normal cluster it was 12%. This underlines that the balance between high psychosocial work stress and recovery is disturbed, especially among the overcommitted shift workers in the risk cluster.

In recent times, research results show how important it is for the regeneration process to be able to “switch off” (20, 26, 27, 55, 94). According to the results from a representative survey among employed persons in Germany (*n* = 4,511, age range: 31–60 years) (94), 12% of shift workers can be expected to have insufficient recovery. In our sample, 16% of all shift workers were impaired in their recovery. Schulz et al. (94) postulate depressive symptoms and sleep disorders as consequences of an inability to recover. According to de Bloom et al. (95), the recovery processes after work make a decisive contribution to maintaining psychological wellbeing and health. For the present study, there was only a very low correlation between psychological wellbeing and the ability to recover (r = 0.18).

Regardless of cluster membership, it is relevant from a preventive perspective that a relatively high proportion of shift workers have cardiovascular risk factors that can lead to health problems or incapacity for work in the medium and long term; for example, for these indicators, more than half of the sample showed hypertonic blood pressure (54%), more than a third (39%) were overweight and a quarter (22%) obese, as well as an increased waist-hip ratio (25%). Furthermore, more than half (57%) of the shift workers stated that they only carried out sporting activities occasionally or not at all, and just under half of the shift workers were smokers (45%). The risk of a heart attack could, however, be classified as low for the majority of shift workers (94%).

In the *DEGS1 study*, if the classic sex and age effects are taken into account, the prevalence of most cardiovascular risk factors are significantly lower than in the present study. In line with this, the age range from 18 to 69 years was also considered in the *DEGS1 studies*. In this age range, a hypertension prevalence of around 24% (men: 30%, women: 19%) is given for the general German population (96). Two thirds of men (67%) and half of women (53%) in Germany are overweight and a quarter of adults (men: 23%, women: 24%) suffer from obesity (79). The higher a person's body fat percentage is above normal, the more dangerous the health consequences are to be expected (97, 98). Obesity in particular leads to increased stress on the musculoskeletal system and promotes the development of lipid metabolism disorders and hypertension (97).

With regard to smoking status, in the *DEGS1 study* was stated that 36% of adults are smokers, with 39% of men and every third woman (33%) in Germany affected (99). Compared to this *DEGS1 study*, significantly more shift workers smoked in our study, namely over half (51%) of the men and 42% of the women. The highest proportion of smokers was recorded in the low-stress cluster (55%), the lowest proportion of smokers in the normal cluster (39%; risk CL: 45%), whereby the shift system effect must be taken into account. The high proportion of smokers is known for hotel and catering industry (66, 100) and shift workers (14, 17, 23, 29).

According to the *DEGS1 study*, about a third (34%) of adults are also inactive in sports, but men are said to be more active, more often (more than 4 hours/week) than women (25 vs. 17%) (101). Although regular physical activity has a positive effect on health at any age, fewer and fewer adults are physically active with increasing age. This affects 15% of 18–29-year-olds, but around half (49%) of those 65 and over (101). In comparison, 43 and 16% of the shift workers stated that they did not or only occasionally exercised. 41% of shift workers reported doing sports regularly. Classification of the results on the basis of the WHO recommendations on health-promoting physical activity was not possible with the available data, but indicates a current field of action for prevention.

The PROCAM Score is of particular importance for the assessment of myocardial infarction risk. Our study confirmed a higher cardiovascular risk for men than for women and this risk increased with age. From the age of 50, 4% of men had a risk >20 and 2% of women had a medium risk of myocardial infarction (10–20%). This means that the risk of myocardial infarction is somewhat lower than in the PROCAM study by Assmann et al. (102), representing the general population in Germany. Also, in the DETECT study by Silber et al. (103), the mean 10-year coronary morbidity risk was estimated to be 4.9% using the PROCAM score and thus higher than in our study. This effect is in contrast with the results of the meta-analysis by Vyas et al. (18), which found that shift workers had a higher cardiovascular disease risk compared with non-shift day workers. However, our shift worker sample is significantly younger (average age: 40 years) than in the comparative studies considered, and the influence of health behavior and social status must be taken into account.

Also, the results of the cardiovascular indicators can be classified contrary to the assumed interaction effect in the ERI model (21). There were no or only very slight correlations between increased psychosocial work stress and health-endangering characteristics of the cardiovascular indicators (R = 0.07–0.13). In the literature reviewed, however, no consistent associations with psychosocial work stress were reported for most cardiovascular indicators either (1, 4). Since more than half (52%) of the shift workers were younger than 40 years of age in our study and cardiovascular restrictions occur more frequently at a later age (104), associations to cardiovascular risk factors may not yet be directly demonstrated. In addition, there is the healthy worker effect (105).

However, neither psychosocial work stress nor shift work necessarily lead to health problems. There are also “healthy” shift workers! More recent studies have addressed the fact that classic cardiovascular risk factors (e.g., lack of exercise, smoking, diet) have a much greater impact on the health of employees than shift and night work (20). According to Struck et al. (106), the health hazards of shift and night work are based more on “third-party variables” (e.g., socio-demographic influencing factors, stressful workplaces) that occur regardless of the working hours. The association between shift and night work and impaired health must be interpreted more cautiously and in a more differentiated manner. Overall, it remains unclear whether shift and night work *per se* lead to health problems, or whether the change in lifestyle and particular personality traits (e.g., excessive willingness to overcommit) are responsible for this, or to what extent the conditions at the workplace (e.g., including social relationships, recognition, employment status) are initially hazardous to health. In addition, the replicability of the clusters in future shift work studies needs to be further examined, as the clusters found resulted from a relatively small sample from two industries.

In summary, for shift work in the hotel and catering sectors and food industries, psychosocial workload seems to be less related to cardiovascular health but more related to psychological health. However, this effect underestimates the considerable health risk for shift workers, which exists due to the health-endangering characteristics of the cardiovascular indicators and signals further need for action in this area. From an occupational health perspective, the prevalence of high blood pressure among shift workers, with an average age of 41, is particularly worrying. There is a clear discrepancy between unknown and treated hypertension since most of the shift workers were not aware of their blood pressure values, nor were they undergoing medical treatment. However, medical therapy to lower blood pressure and thus avoid secondary diseases caused by hypertension is only given to diagnosed patients. To this end, occupational health care are not used sufficiently.

The originality of the study is that the interaction hypothesis of the *ERI model* (21) was tested for the first time in shift workers in hotel and catering and the food industry using a three-cluster solution. Another special feature that must be emphasized is that the blood pressure status from a 4-day self-measurement was generated from 24 measured values according to the guidelines of the European Society of Cardiology and the European Society of Hypertension (78) and the criteria for the diagnosis of “hypertension” were met. Thirdly, with the characteristic of inability to recover in the context of the *ERI model* (21), a new aspect that had not been investigated was introduced into this model.

### Limitations of the Study

When interpreting the results, the following limitations of this study must be taken into account:

#### Sample

Since participation in the study was voluntary and it was therefore an convenience sample, selection effects can also exist. Due to the age of the sample and the known drop-out rate in three-shift systems with night work and in the hotel and catering sector, a healthy worker effect cannot be ruled out, which can lead to an underestimation of the health risks.

#### Research Design

As the data were collected as part of the cross-sectional design, no statements can be made on the cause-and-effect relationship. But reverse causality should be considered. Shift workers who have health problems may report more stress.

#### Questionnaires

Some of the variables were recorded by questionnaires or self-assessment. Data collected in this way are subject to known quality restrictions (e.g., distortion due to social desirability, response tendencies, memory deficits, recall bias). This method-critical objection was largely remedied by including objective health indicators (e.g., self-measurement of blood pressure, measurement of body dimensions). This is also one of the strengths of this investigation.

#### Health Behavior

Alcohol consumption, although a specific health characteristic in hotel and catering industry and may be associated with increased psychosocial stress, was excluded from the analyses because the data often reflect a bias due to social desirability.

#### ERI-Q

The model structure of the ERI-Q (21) was checked in several validation studies (107–109), whereby a factor-analytically unclear structure and no uniform operationalisation were found for the overcommitment component (OC) in some studies. The OC construct (21) is also described in the literature as a lack of ability to distance oneself from work (61, 109–111), and striving for perfection (112).

## Conclusion

The results of the three-cluster solution signal the need for occupational health prevention and care. They contribute to the assessment of the burden of illness through psychosocial work stress among shift workers. The significance of psychosocial work stress in combination with severe overcommitment is only confirmed in the present study by the data on psychological health. However, this result underestimates the considerable health risk for shift workers, which exists due to the health-endangering characteristics of the cardiovascular indicators. Men are particularly affected here, while psychological health impairments are more prevalent among women, and especially those who worked in the alternating shift system with night shifts.

Preventive occupational health programmes represent a suitable approach to detecting endangered shift workers or risk groups at an early stage. In addition to health behavior, the job characteristics themselves, the working conditions and the design of the shift or working time models are among the preventive starting points for company medical practices and occupational health screening concepts for shift workers. Even if the cardiovascular indicators could not contribute to the explanation of the ER-OC clusters in the present study, it is necessary to integrate these indicators into a precautionary concept for shift workers, in order to be able to estimate the cardiovascular risk of the employees. These concepts should be supplemented by the topics of psychosocial work stress and the ability to recover. Both constructs have achieved a new status in the modern working world, but many new questions still need to be clarified in future research. Our results support the fact that recovery has an independent diagnostic value for assessing work stress. It is interpreted as an early indicator for the detection of psychosocial work stress in above-average committed shift workers. It is crucial to identify and influence psychosocial work stress and health hazards as well as work and health resources at an early stage.

In order to cope with the high work stress caused by shift and night work, a balance between work and recovery and sufficient regeneration phases are an essential prerequisite for high performance. A high level of effectiveness and productivity at work is only possible if the employees succeed in fully regenerating their performance requirements. Since ability to recover has been shown to be the most important predictor for explaining the psychosocial work stress, it should be definitely included in future shift work studies.

## Data Availability Statement

The raw data supporting the conclusions of this article will be made available by the authors, when a reasoned request is made to the authors. There is an agreed data protection obligation towards the participants.

## Ethics Statement

The studies involving human participants were reviewed and approved by Ethics Committee of the Technical University Dresden (EK 250397). The participants provided their written informed consent to participate in this study.

## Author Contributions

RS and BH made the funding acquisition. RS designed the study, performed the statistical analyses and interpretation of the data, and wrote the first draft of the manuscript. Both authors made the project administration and collected the data. BH commented critically on the manuscript. Both authors approved the submitted version.

## Funding

As a matter of legal succession, this work was funded by the German Social Accident Insurance Institution for the Foodstuffs and Catering Industry (BGN) in Mannheim.

## Conflict of Interest

The authors declare that the research was conducted in the absence of any commercial or financial relationships that could be construed as a potential conflict of interest.

## Publisher's Note

All claims expressed in this article are solely those of the authors and do not necessarily represent those of their affiliated organizations, or those of the publisher, the editors and the reviewers. Any product that may be evaluated in this article, or claim that may be made by its manufacturer, is not guaranteed or endorsed by the publisher.

## Abbreviations

BGN: German Government Safety Organization Foods and Restaurants
CL: cluster
ArbZG: Working Hours Act
FI: food industry
HCI: hotel and catering industry
ER: effort-reward
ERI: effort-reward imbalance
OC: overcommitment
IR: inability to recovery
BP: blood pressure
BMI: body mass index
WC: waist circumference
WHR: waist-hip ratio
DBP: diastolic blood pressure
SBP: systolic blood pressure
PROCAM: Prospective Cardiovascular Münster
SPSS: Statistical Package for the Social Science
ESH: European Society of Hypertension
ESC: European Society of Cardiology
ERI-Q: Effort-Reward Imbalance Questionnaire
GHQ-12: General Health Questionnaire-12
FABA: Questionnaire for Faulty Attitudes and Behavioral Analysis Relevant to Coping with Work Demands
DEGS1: German Health Interview and Examination Survey for Adults.
